# MESP2 binds competitively to TCF4 to suppress gastric cancer progression by regulating the SKP2/p27 axis

**DOI:** 10.1038/s41420-023-01367-4

**Published:** 2023-03-01

**Authors:** Lingjun Ge, Gaichao Zhao, Chao Lan, Houji Song, Dan Qi, Pan Huang, Xiaoxue Ke, Hongjuan Cui

**Affiliations:** 1 State Key Laboratory of Silkworm Genome Biology, Southwest University, Chongqing, 400716 China; 2 Cancer Center, Medical Research Institute, Southwest University, Chongqing, 400716 China

**Keywords:** Tumour-suppressor proteins, Cell growth, Cell invasion

## Abstract

Gastric cancer (GC) is a major cause of human deaths worldwide, and is notorious for its high incidence and mortality rates. Mesoderm Posterior Basic Helix-loop-helix (bHLH) transcription factor 2 (MESP2) acts as a transcription factor with a conserved bHLH domain. However, whether MESP2 contributes to tumorigenesis and its potential molecular mechanisms, remain unexplored. Noticeably, MESP2 expression levels are decreased in GC tissues and cell lines compared to those in normal tissue. Further, in vitro and in vivo experiments have confirmed that MESP2 overexpression suppresses GC cell growth, migration, and invasion, whereas MESP2 knockdown results in the exact opposite. Here, we present the first report that MESP2 binds to transcription factor 7-like 2 (TCF7L2/TCF4) to inhibit the activation of the TCF4/beta-catenin transcriptional complex, decrease the occupancy of the complex on the S-phase kinase Associated Protein 2 (SKP2) promoter, and promote p27 accumulation. MESP2 knockdown facilitated tumorigenesis, which was partially suppressed by SKP2 knockdown. Taken together, we conclude that MESP2 binds competitively to TCF4 to suppress GC progression by regulating the SKP2/p27 axis, thus offering a potential therapeutic strategy for future treatment.

## Introduction

Gastric cancer (GC), a deadly malignancy, is the third main cause of global human cancer-related deaths, resulting in ~723000 deaths annually [1]. Nearly half of the cases occur in Eastern Asia, especially in China [1, 2]. Clinically, early disease development is rarely accompanied by GC symptoms [3], and patients have poor rates of survival, mainly due to suboptimal treatment and late diagnosis [1]. There is therefore, an urgent need for elucidating the potential molecular mechanisms of GC progression and determining effective therapeutic strategies.

Mesoderm posterior 2 (MESP2), is a transcription factor with a basic helix–loop–helix (bHLH) motif that has been illustrated to play pivotal roles during the development of mammalian somite and heart. MESP2 links spatiotemporal information, produced by the determination wave front and the segmentation clock, to multiple somite morphogenesis processes in mouse somitogenesis [4]. The controlled segmentation program of the Notch signalling pathway reflects the important function of MESP2, and the determination of the segment boundary of the Wnt3a/beta-catenin signalling pathway was demonstrated by an MESP2 expression assay of Wnt3a mutants [5]. In fact, boundary formation and somite compartmentalization seem to depend on the combined action of Wnt and Notch signalling. Interrupting either pathway restrains somite formation and the MESP2 protein levels that indicate somite compartmentalization and boundary formation [6]. The bHLH family is one of the most prominent among transcription factors [7] and is involved in cell differentiation and tissue development [8]. These TFs share a characteristic protein structure composed of a basic region [9] that interacts with DNA and a neighboring helix–loop–helix region that mediates dimerization [10]. Most bHLH dimers recognize the E-box, a hexameric sequence in the DNA with the consensus sequence CANNTG [10]. These bHLH TFs modulate gene expression through dimer formation, combining activators or repressors with ubiquitous proteins (E-proteins) [11]. Previous experiments have showed that aberrant expression of the bHLH transcription factor is related to tumorigenesis [12], suggesting that MESP2 may also participate in human cancer progression.

The Wnt signalling pathway influences the regulation of intestinal development, differentiation, and adult tissue homeostasis, thus contributing to the progression of numerous human cancers [13]. Transcription factor 7-like 2 (TCF7L2/TCF4) is a key component of the Wnt signalling pathway, mostly acting as a transcription factor after entering the nucleus [14]. Furthermore, Wnt ligands trigger the typical Wnt/beta-catenin signalling pathway, which in turn activates the LRP and Frizzled receptors, leading to beta-catenin stabilization and nucleus translocation. Subsequently, nuclear beta-catenin binds to TCF4, thereby promoting the expression of relevant genes [15]. In the intestinal epithelium, the expression levels of TCF4/beta-catenin downstream target genes are regulated by mutational activation of Wnt signalling, then affecting malignant transformation of colorectal cancer [13]. The ultimate aim of the typical Wnt signalling in the beta-catenin pathway is the formation of the TCF4/beta-catenin complex, and this interaction is regarded as a valid target for cancer treatment [16].

The F-box protein S-phase kinase-associated protein 2 (SKP2) is a subunit that recognizes substrates of the SCF–SKP2 E3 ligase, targeting protein proteasome-mediated degradation, such as p27 [17]. The deletion of p27 and tumorigenesis often go hand-in-hand. The influence of p27 in tumorigenesis is supported by the formation of spontaneous pituitary tumors in p27^−/−^-mice [18]. Functionally, the CDK suppressor, p27, is a typical negative regulator of cell proliferation. It can inhibit the G1-S transition of cyclinE/CDK2 complex and the S phase of cyclinA/CDK2 complex [19]. Physiological regulation of G1-S transformation is crucial in determining cell fate and is lost during oncogenic transformation. In addition, in a range of cancer types, the declined cytoplasmic p27 protein expression is pertinent to high metastasis and poor survival. The loss of p27 affects self-renewal of hematopoietic stem cells and the proliferation and differentiation of hematopoietic progenitor cells, making cells prone to tumor transformation [20].

In this work, considering that MESP2 expression levels are reduced in GC tissues and cell lines, we focused on MESP2 regulation and function, which to our knowledge, are uninvestigated in cancer. Functionally, our study revealed that MESP2 binds competitively with beta-catenin to TCF4, suppressing GC progression by regulating the SKP2/p27 axis. Thus, MESP2 is a candidate in the diagnosis and therapy of GC.

## Results

### MESP2 as a diagnostic/prognostic marker for GC

**Fig. 1:**
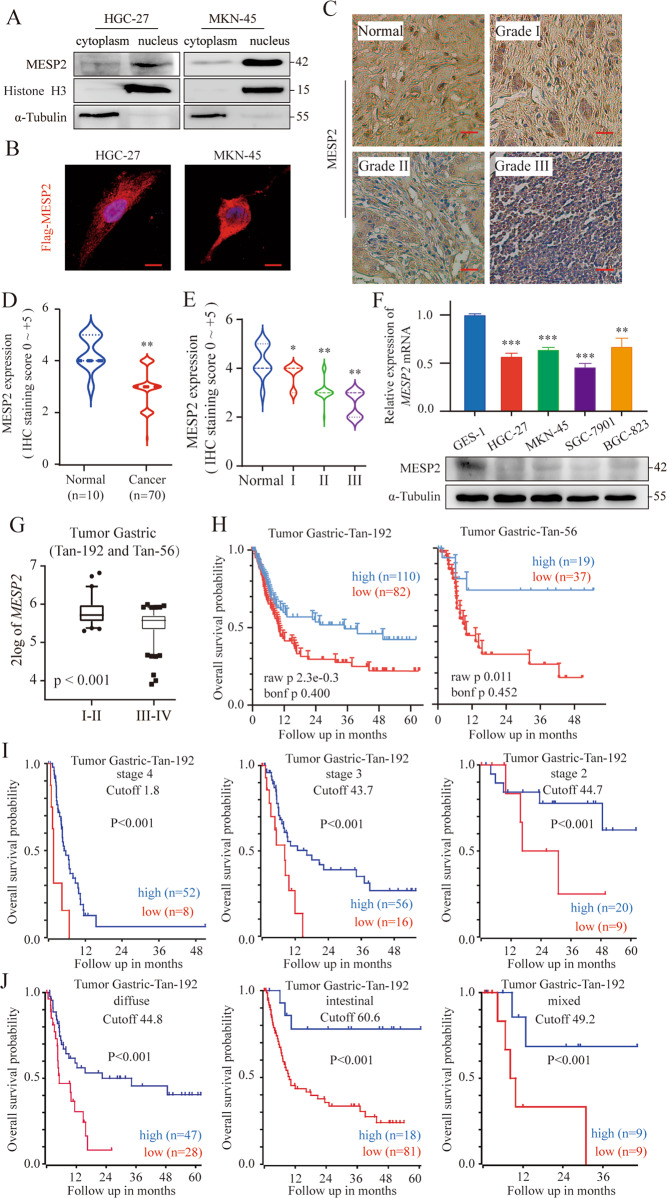
MESP2 expression is downregulated in GC tissues and related to high prognosis. **A** Subcellular fractionation assays display MESP2 levels. Cytoplasmic α-tubulin and nuclear Histone H3 were used as reference standards. **B** Intracellular localization analysis of Flag-MESP2 by immunofluorescence staining. Intracellular localization of Flag-MESP2 (red); Nuclei (blue) were stained with DAPI. Scale bar, 5 μm. **C** Representative images of MESP2 expression at different levels in gastric tumor (I, II, III) and normal tissues. Scale bar, 20 μm. **D**, **E**. IHC staining analyses of MESP2 expression levels in 80 samples of normal tissues and gastric tumor tissues. ***P < 0.001. **F** qRT-PCR and Western blot experiments showing MESP2 mRNA and protein levels in GC cell lines (HGC-27, MKN- 45, SGC-7901, BGC-823, GES-1). Data shown are means ± SD, *n* = 3, **P < 0.01, ***P < 0.001. **G** Box plot of MESP2 expression levels in GC tissues in the R2 database. **H** Kaplan-Meier analysis of progression-free survival using data from R2 databases with the log-rank test *P*-values indicated. **I**, **J**. Kaplan-Meier analysis of progression-free survival was performed for patients with different grades and subtypes of GC using data from R2 databases with the log-rank test *P*-values indicated.

To explore the functional importance of MESP2 in GC, we first examined the intracellular localization of MESP2 in GC cells. The findings showed that it was located in both the cytoplasm and nucleus (Fig. 1A, B). Next, we analyzed MESP2 levels in 80 tissue samples of clinical patients via IHC staining. MESP2 IHC staining was notably lower in gastric tumor tissues in a grade-dependent pattern compared to that in normal tissues (Fig. 1C–E). qRT-PCR and western blot experiments showed that mRNA and protein expression of MESP2 were downregulated in HGC-27, MKN-45, SGC-7901 and BGC-823, contrasted with that of GES-1, a normal human gastric epithelial cell (Fig. 1F). Moreover, Analysis of MESP2 expression levels in GC patient tumors using the R2: Genomics Analysis and Visualization Platform (https://hgserver1.amc.nl/cgi-bin/r2) consistently confirmed that MESP2 upregulation predicted better patient survival (Fig. 1G–J). Taken together, our results indicate that *MESP2* may be an anti-oncogene.

### Overexpression of MESP2 suppressed GC progression in vitro and in vivo, but downregulation resulted in the opposite

To further investigate the impact of MESP2, GO analysis showed that differentially expressed genes were mainly enriched in the biological regulation and by the molecular function regulator (Supplementary Fig. 1A).

**Fig. 2:**
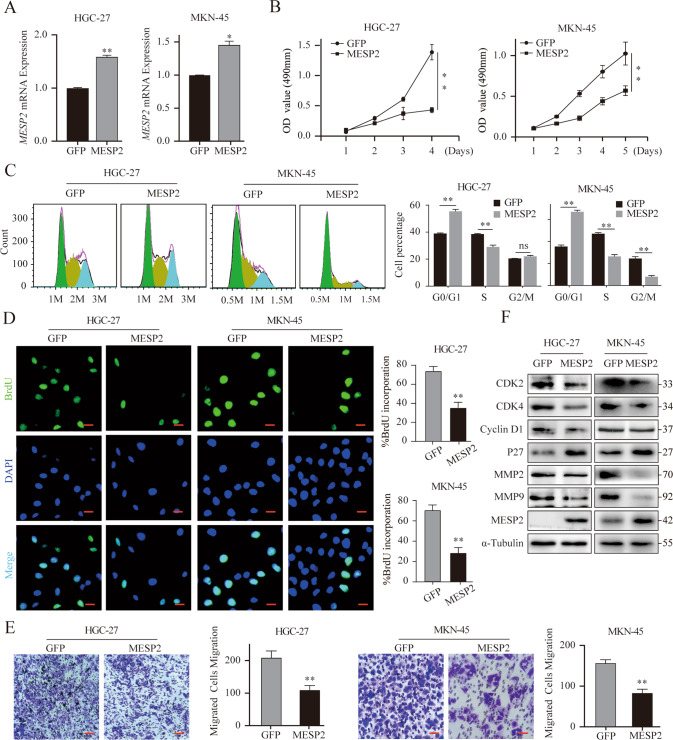
MESP2 inhibits the proliferation, migration and invasion ability of GC cells both in vitro and in vivo. **A** qRT-PCR analysis of MESP2 mRNA levels in MESP2-overexpression cells. **B** MTT assays showing suppressed GC cell proliferation by MESP2 overexpression. **C** The HGC-27 and MKN-45 cell cycles were detected via flow cytometry after overexpression of MESP2. **D** Representative fluorescent images and quantification of BrdU staining of GC cells expressing GFP or MESP2. Scale bar, 20 μm. **E** The migratory capacity of GC cells transfected with GFP or MESP2, as determined via transwell assays. Scale bar, 20 μm. Data shown are means ± SD, *n* = 3, *P < 0.1, **P < 0.01. **F** Western blots showing the levels of G1 phase regulatory and pivotal transfer-associated proteins.

**Fig. 3:**
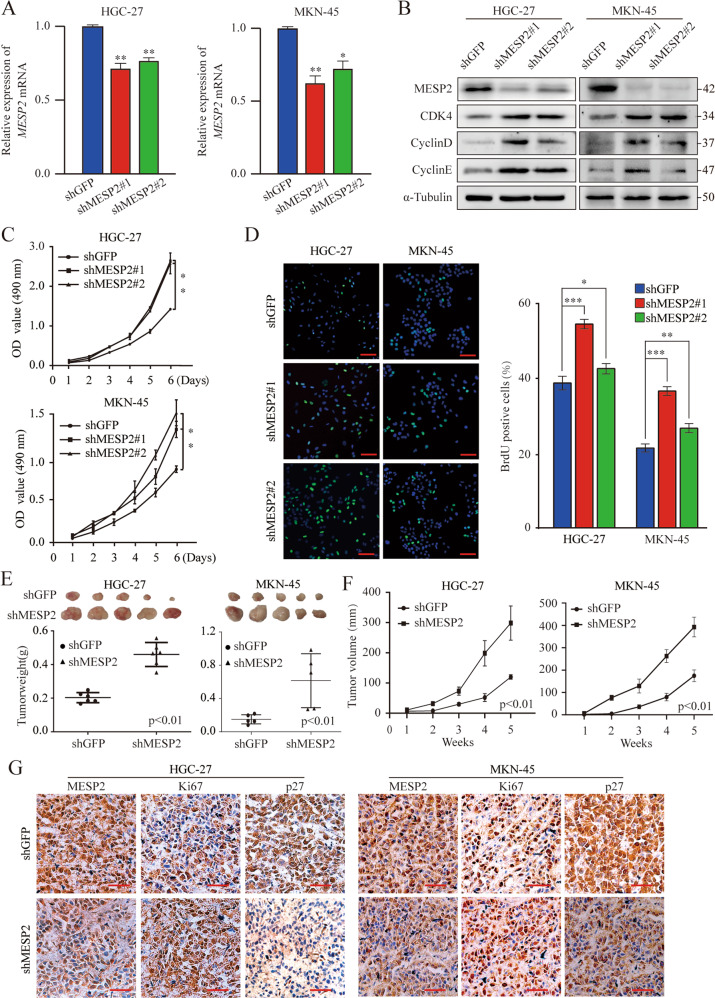
Knockdown MESP2 promotes the proliferation ability of GC cells both in vitro and in vivo. **A** qRT-PCR was performed to assess MESP2 mRNA levels in MESP2-knockdown cells. **B** Western blot experiments showing G1 phase regulatory protein levels in MESP2-knockdown cells. **C**. MTT assays showing that MESP2 knockdown promoted GC cell proliferation. **D** Representative fluorescent images and quantification for BrdU staining of GC cells expressing shGFP, shMESP2#1, or shMESP2#2. Scale bar, 20 μm. **E**, **F** In vivo analyses of size and volume of xenograft tumors that were hypodermically injected with treated HGC-27 and MKN-45 cells. **G** Representative IHC micrographs of selected tumors. Scale bar, 20 μm. Data shown are means ± SD, *n* ≥ 3, *P < 0.05, **P < 0.01, ***P < 0.001.

Then we successfully overexpressed MESP2 in in HGC-27 and MKN-45 cells lines (Fig. 2A). Besides, knocked down MESP2 expression using the lentiviral shRNA, namely, shMESP2 nos. 1 and 2 (Fig. 3A, B). In MTT assays, MESP2 overexpression substantially suppressed cell growth (Fig. 2B). Conversely, knockdown of MESP2 promoted GC cell proliferation, compared with control cells (Fig. 3C). To further examine the molecular mechanism of MESP2-mediated inhibition of cell growth, we utilized flow cytometry to determine the effect of MESP2 on cell cycle progression. We showed that MESP2 overexpression arrested the cell cycle at the G0/G1 phase (Fig. 2C). Subsequently, BrdU staining signified that BrdU-positive rates in MESP2- upregulated cells were reduced (Fig. 2D). The changes noted in the knockdown group were different (Fig. 3D). Consistent with this finding, the levels of critical G0/G1 regulon genes, such as *CDK4*, *CCND1*, and *CCNE1*, decreased with MESP2 upregulation, whereas knockdown of MESP2 led to the reverse (Fig. 2F, Fig. 3B, Supplementary Fig. 1B). We used a mouse xenograft model to verify the putative tumor suppressor role of MESP2 in vivo, and discovered that knockdown of MESP2 promoted tumor growth and volume (Fig. 3E, F). Furthermore, IHC staining implied that tumors formed by MESP2 knockdown cells exhibited higher Ki67 and lower p27 staining than those formed by control cells (Fig. 3G, Supplementary Fig. 1C).

**Fig. 4:**
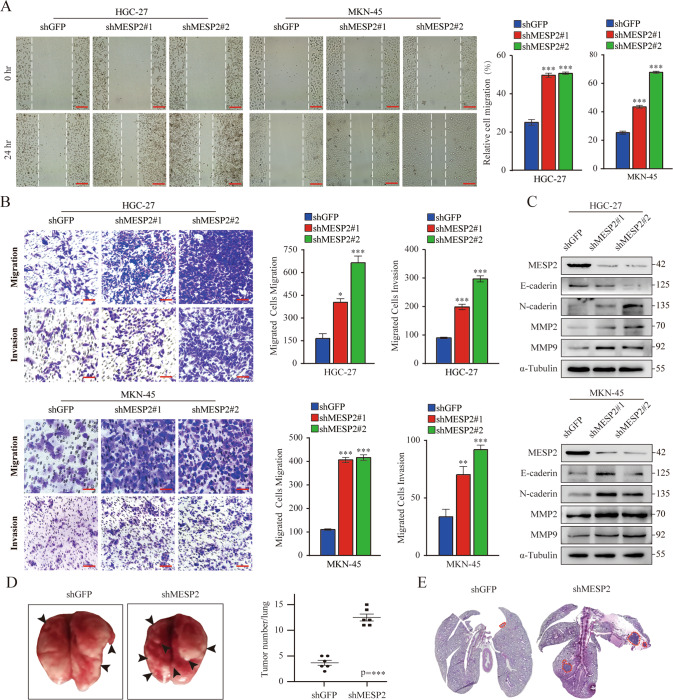
Knockdown MESP2 promotes the migration and invasion of GC cells in vitro and in vivo. **A**, **B** The migration and invasion capacities of GC cells transfected with shGFP alone, shMESP2#1 or shMESP2#2 were determined via Wound healing and transwell assays. Scale bar, 20 μm. **C** Western blot experiments showing the levels of several pivotal transfer-associated proteins. **D** Images showing that the shMESP2 mouse model had more pulmonary nodules (black arrows) than the others. **E** Histological analysis of pulmonary tumors as observed via H&E staining are shown. Scale bar, 2 mm. Data shown are means ± SD, *n* ≥ 3, *P < 0.05, **P < 0.01, ***P < 0.001.

After assessing the functions of MESP2 in GC cell growth, we confirmed its function in metastasis. Transwell assays demonstrated that migration and invasion behaviors were significantly inhibited by MESP2 overexpression (Fig. 2E), but enhanced by knockdown of MESP2 (Fig. 4B). Wound-healing assays also confirmed the above results (Fig. 4A). Western blot experiments using epithelial-mesenchymal transition (EMT)-related markers and matrix metalloproteinases (MMPs), displayed same trends. Upregulation of MESP2 decreased MMP2 and MMP9 levels (Fig. 2F). Nevertheless, Knockdown of MESP2 reduced E-cadherin protein levels, but upregulated N-cadherin, and MMPs protein levels (Fig. 4C, Supplementary Fig. 1B). Subsequently, we assessed the influence of MESP2 on lung metastasis. The number of pulmonary metastatic nodules in MESP2 downregulation group greatly increased in comparison to the control group (Fig. 4D). Hematoxylin and eosin (HE) staining revealed that treatment with shMESP2 effectively promoted tumor cell metastasis (Fig. 4E). Collectively, these results strongly imply that MESP2 can serve as a potential tumor suppressor in GC.

### MESP2 induced the stabilization of p27 by inhibiting its ubiquitylation and subsequent degradation

**Fig. 5:**
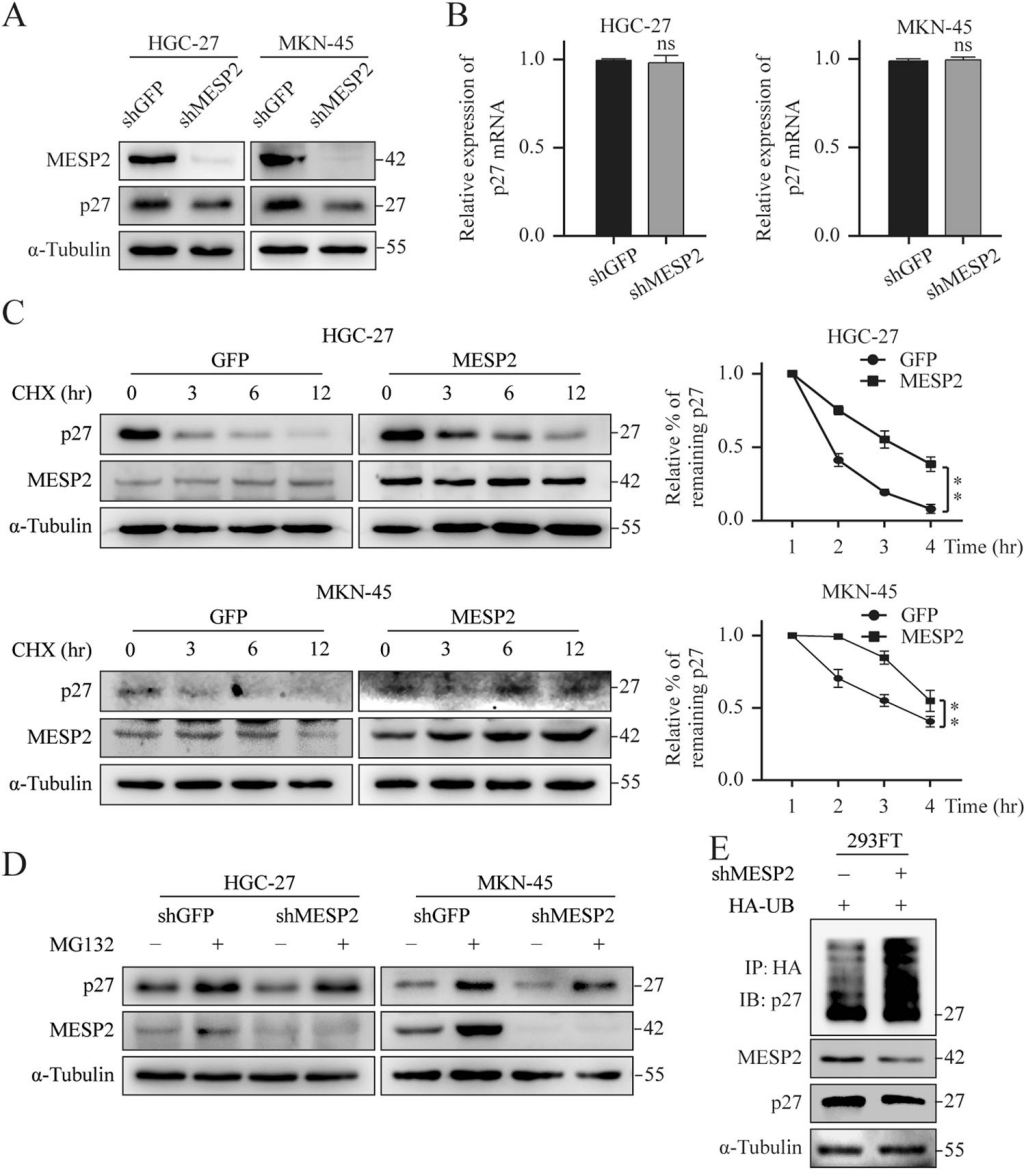
MESP2 regulates p27 ubiquitination and degradation. **A**, **B** protein and mRNA levels of p27 in MESP2-knockdown cells were examined via western blotting and qRT-PCR. **C**. Western blots of cells overexpressing GFP or MESP2 after treatment with CHX (100 μg/ml) for a specific period. **P < 0.01. **D** GC cells with shGFP or shMESP2 were dealt with vehicle (−) or MG-132 (50 μg/ml) about 6 h prior to being immunoblotted for the protein expression levels of p27. **E** Effect of MESP2 knockdown on p27 ubiquitination in 293FT cells.

Meanwhile, we observed that MESP2 downregulation decreased the protein levels of p27 (Fig. 5A), which acted as a CDK inhibitor and controlled cell proliferation and metastasis [21]. Multiple studies, including our previous research, have showed that p27 can combine and suppress the activity of the cyclin E/CDK2 complex to block the G1 phase [22]. To further elucidate the regulatory mechanisms of MESP2-stabilizing p27 expression, p27 mRNA levels in the experimental and control groups were assessed via qRT-PCR. Contrary to p27 protein levels, MESP2 knockdown did not impact p27 mRNA levels significantly (Fig. 5B). Therefore, we speculated that a post-transcriptional mechanism was involved in the changes in p27 expression levels. We evaluated the protein stability of p27 by introducing a cyclohexamide (CHX), and found that MESP2 overexpression significantly lengthened the half-life of p27 (Fig. 5C), revealing that the p27 expression is positively regulated by MESP2. Knockdown of MESP2 induced p27 degradation and was blocked by Proteasome inhibitors MG-132 treatment (Fig. 5D), supporting the ubiquitination-proteasome pathway to maintain p27 protein levels. Additionally, the ubiquitylation assay showed that downregulation of MESP2 enhanced the poly-ubiquitylation of p27 (Fig. 5E). Finally, transcriptome profiling showed that MESP2 depletion suppressed the p27 pathway (Supplementary Fig. 2A). Based on these results, we concluded that MESP2 indeed improved the protein stability of p27 by suppressing its ubiquitylation and subsequent degradation.

### MESP2 inhibited activation of the TCF4/beta-catenin transcriptional complex by competing with beta-catenin for binding TCF4

**Fig. 6:**
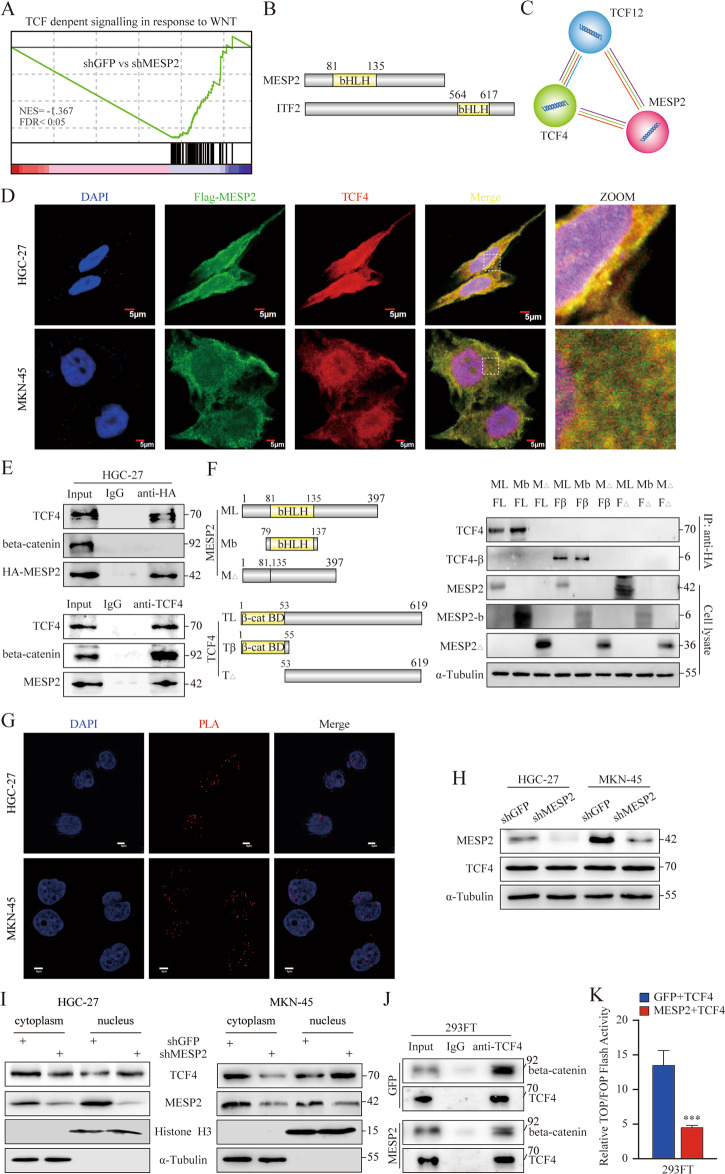
MESP2 binds competitively to TCF4 to inhibit activation of the TCF4/beta-catenin transcriptional complex. **A** A plot of the TCF-dependent signalling in response to the Wnt pathway from gene set enrichment analysis (GSEA) of genes negatively correlated with MESP2 expression. **B** Schematic diagrams of the bHLH domain organization for human MESP2 and ITF2. **C** A prediction of the interaction between the MESP2 and TCF4 was made using the STRING website. **D** Immunofluorescence co-localization staining of Flag-MESP2 and TCF4 in HGC-27 and MKN-45 cells. Scale bar, 5 μm. **E** Interaction of endogenous MESP2 with endogenous TCF4. **F** Mapping of TCF4-binding domains on MESP2. HA-MESP2 (aa 1–397), HA-Mb (aa 79–137), HA-MΔ (aa 1–79,137–397), Flag-TCF4 (aa 1–619) Flag-T1 (aa 1–55) and Flag-TΔ (aa 56–619) were co-transfected in pairs into 293FT cells. **G** Proximity ligation assay indicating the interaction of MESP2 and TCF4 in HGC-27 and MKN-45 cells (red: PLA positive signal; blue: DAPI, scale bar = 5 μm). **H** TCF4 levels in MESP2-knockdown HGC-27 and MKN-45 cells. **P < 0.01. **I** Subcellular fractionation assays display TCF4 levels. Cytoplasmic α-tubulin and nuclear Histone H3 were used as reference standard. **J** Co-IP assays were used to examine TCF4 binding to beta-catenin after MESP2 overexpression. **K** Wnt-reporter luciferase activity in 293FT cells with or without MESP2 depletion and TCF4 overexpression. ***P < 0.001.

We further characterized the biological functions of MESP2 and deciphered the mechanisms of its roles in GC inhibition via transcriptome profiling, which revealed that MESP2 depletion activated TCF-dependent signalling in response to the Wnt pathway (Fig. 6A). The known TCF4/beta-catenin transcriptional complex has great effects on the Wnt pathway. Through functional domain analyses, Studies have reported that ITF2 and MESP2 exhibited the relatively well-conserved bHLH domain (Fig. 6B), and a previous study had showed that ITF2 suppressed formation of the TCF4/beta-catenin complex by competing with beta-catenin for TCF4 [13]. Hence, we assessed through an online prediction website whether MESP2 and TCF4 had a binding relationship (Fig. 6C). We further validated whether both proteins integrated with each other under experimental conditions, and immunofluorescence staining showed that MESP2 was co-expressed with TCF4 in HGC-27 cells (Fig. 6D). Then, co-IP assays showed that TCF4 was immunoprecipitated by MESP2 (Fig. 6E). To gain further insight into the specific domains that mediated the interaction between TCF4 and MESP2, we truncated the full length of TCF4 into a T1 fragment with beta-catenin binding. Similarly, the full-length MESP2 was truncated into an Mb fragment with the bHLH domain (Fig. 6F). Next, for domain-mapping experiments, we transfected 293FT cells with Flag-TCF4 (amino acids (aa) 1–619) or Flag-T1 (aa 1–55) or Flag-TΔ (aa 56–619) of TCF4 and immunoprecipitated these against HA to observe their interaction with Flag-TCF4 or Flag-T1 or Flag-TΔ in vivo. Both TCF4 and T1 bound to MESP2 or M1 (Fig. 6F).

We detected no further significant changes in the total expression levels of TCF4 in MESP2-knockdown cells (Fig. 6H). It is well known that the ability of the transcription factor to perform its role always relies on transport between the cytoplasm and nucleus, which is controlled by specific sequences. Impressively, the bHLH domain of TCF4 contains specific sequences i.e. a nuclear localization signal that overlaps two nuclear export signals [23]. Consequently, we found that the amount of TCF4 in the nuclear fraction increased remarkably after knocking down MESP2 (Fig. 6I). Mutational activation of Wnt signalling aberrantly transactivates downstream TCF4/beta-catenin target genes, further contributing to malignant transformation in human cancers. In addition, we examined that MESP2 overexpression drastically decreased direct binding between beta-catenin and TCF4, on the basis that MESP2 did not bind to beta-catenin (Fig. 6J). Luciferase reporter assay showed that TCF4 activity decreased after overexpression of MESP2 (Fig. 6K). Altogether, these observations support the proposal that MESP2 competes with beta-catenin for binding to TCF4 to inhibit activation of the TCF4/beta-catenin transcriptional complex.

### MESP2 overexpression lessened the occupancy of TCF4/beta-catenin on SKP2 promoter, leading to subsequent accumulation of p27

**Fig. 7:**
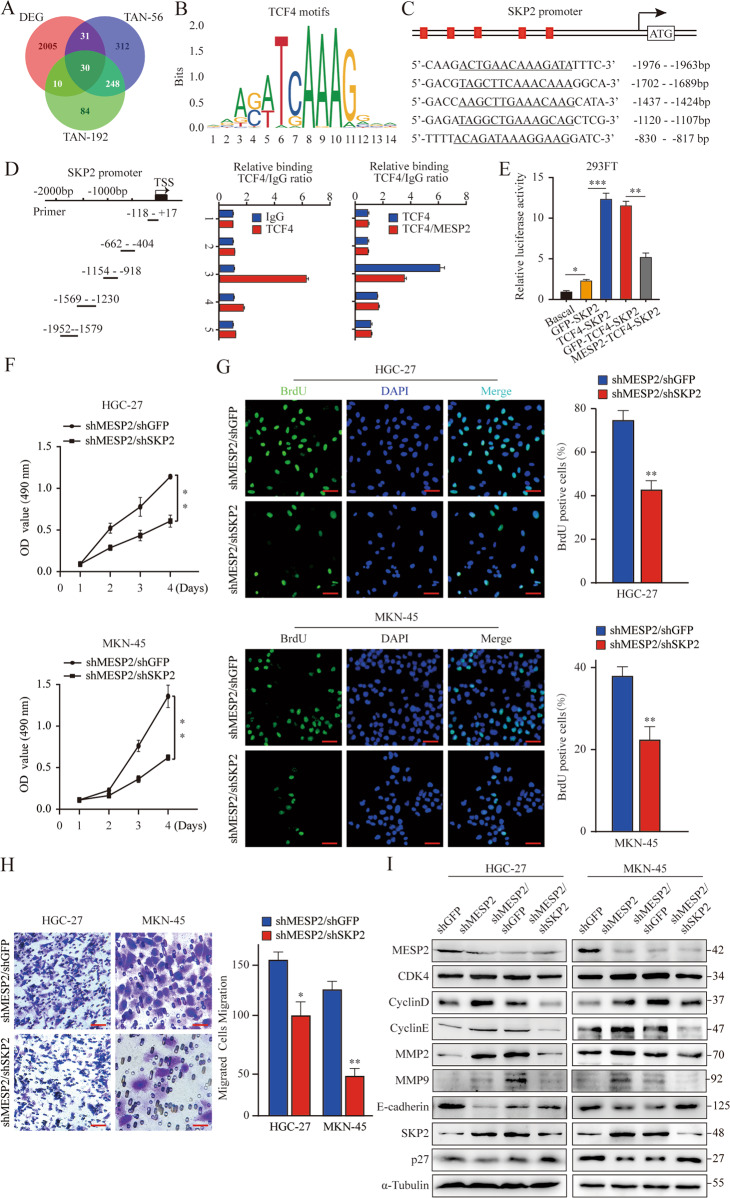
The depletion of MESP2 enhances the occupancy of TCF4/beta-catenin on the SKP2 promoter. **A** Venn diagram indicating 30 differential genes identified in all three cohorts. **B** JASPAR analysis of the DNA-binding motifs of TCF4. **C** Identification of TCF4 binding site in the SKP2 promoter. **D** Five primer sets were designed within the human SKP2 promoter. A Chromatin immunoprecipitation assay was carried out using Flag antibody. IgG was the negative control. **E** Luciferase activity was examined 48 h post-transfection. The pGL3-basic vector was used as the negative control. **F** MTT assays showing the proliferation ability of GC cells with knockdown of both MESP2 and SKP2. **G** Representative fluorescent images and quantification for BrdU staining of GC cells expressing shMESP2/shGFP or shMESP2/shSKP2. Scale bar, 20 μm. **H** Transwell assays showing the migration ability of GC cells with knockdown of both MESP2 and SKP2. Scale bar, 20 μm. **I** Western blots showing the levels of proliferation and transfer-associated proteins mentioned above. Data shown are means ± SD, *n* = 3, *P < 0.05, **P < 0.01, ***P < 0.001.

To narrow down potential MESP2 migratory targets that are relevant across GC, we analyzed the intersection between MESP2-KD DEGs and migratory sample DEGs (GSE15459 and GSE34942). We found 30 overlapping genes. GO function enrichment analysis for DEGs were performed using the DAVID (Fig. 7A). The results of GO function enrichment analysis for DEGs indicated that DEGs were mainly enriched in post−translational protein modification and extracellular matrix (ECM). Interestingly, for the cell component, the DEGs were enriched in SCF ubiquitin ligase complex (Supplementary Fig. 2B).

Previous studies have demonstrated that SKP2 is a major SCF ubiquitin ligase that regulates p27 ubiquitination [17]. Thus, we speculate that the accumulation of p27 by overexpressing MESP2 may be due to the inhibition of SKP2 transcription mediated by the TCF4/beta-catenin complex. To identify which nucleotides and region are important in determining SKP2 transcription, bioinformatics analyses using the promoter 2.0 prediction server (http://www.cbs.dtu.dk/services/promoter/), JASPAR (http://jaspar.genereg.net/) were performed to identify the DNA bindings motifs of TCF4 (Fig. 7B). Further analyses of TCF4 binding sites revealed that the promoter of SKP2 contains four binding motifs in a relatively concentrated and overlapped region (Fig. 7C). Then, we sought to identify whether MESP2 could affect the association of TCF4 with the SKP2 promoter. As expected, TCF4 could bind to the SKP2 promoter. Importantly, MESP2 overexpression significantly inhibited the association of TCF4 with the SKP2 promoter (Fig. 7D). Next, luciferase reporter assay showed that transfection of TCF4 increased the luciferase activity of the SKP2 reporter in comparison with that in the control group (Fig. 7E). Impressively, the luciferase activity of the SKP2 reporter was notably inhibited after co-transfection of MESP2 and TCF4 (Fig. 7E). Finally, we found that knockdown of SKP2 and MESP2 suppressed the proliferation and migration of GC cells (Figs. 7F–I), which further ascertained that MESP2 regulated p27 ubiquitination through SKP2. Consistently, these related protein expression levels were partially inhibited (Fig. 7I). Similarly, there was a similar trend in the patient database, although it did not show a significant difference (Supplementary Fig. 2C).

## Discussion

Although investigators have achieved some success in treating patients with GC, there are currently strict criteria for determining the prognosis of patients with GC [24]. Hence, targeting tumor suppressors in GC is a promising therapeutic strategy. Herein, we detected for the first time, a decline in MESP2 expression in gastric tumor tissues that were simultaneously negatively correlated with GC grade. Consistent with this result, MESP2 upregulation was intensively correlated with better prognosis, suggesting that MESP2 may play a role in the inhibition of GC progression. It is now becoming firmly established that MESP2 levels show signs of a periodic pattern that is necessary for the formation of segmental borders [25]. Other studies have demonstrated heritable mutations of MESP2 in human patients with heritable axial skeleton growth disorder [26]. Nevertheless, the function of MESP2 in human cancers have not been characterized till date.

Next, we found that a reduction in MESP2 expression levels increased proliferative and metastatic behaviors, and upregulation of MESP2 decreased relevant behaviors of GC cells. Indeed, MESP2 overexpression strikingly inhibited GC cell proliferation via a G1 phase arrest, the cell cycle was regulated by cyclin-dependent kinases, and the inhibitor p27 suppressed cell proliferation by hampering the cell cycle [27]. Accumulated evidence shows that elevated p27 expression eventually causes growth inhibition in various human cancers [28]. We noticed a decrease in p27 levels, but p27 mRNA levels did not change after knockdown of MESP2. Reduced p27 expression is correlated with high rates of recurrence and metastasis in a variety of malignancies. As documented, the p27 expression status is a key independent prognostic element in patients with GC [29]. Given that the impact of p27 can be disrupted in human cancers by excess proteolysis, C-terminal phosphorylation, or reduced translation, we found that deletion of MESP2 leading to p27 downregulation was due to SKP2-mediated ubiquitination through the proteasome pathway. Meanwhile, knockdown of SKP2 partially suppressed the growth and metastasis of GC cells enhanced via downregulation of MESP2.

Furthermore, we provided evidence for the potential anti-cancer application of MESP2 as a marker for targeted therapy involving TCF4/beta-catenin signalling. After knockdown of MESP2, we found that the activity of theTCF4/beta-catenin transcription complex via the Wnt pathway and the expression of its downstream target genes (*MMPs*) increased in GC cells. The Wnt pathway dominates cell proliferation, cell differentiation, and EMT, which play increasingly crucial roles in human cancers [30]. Based on these results, we present a new hypothesis that TCF4/beta-catenin signalling is controlled inhibited by MESP2, but becomes significantly activated in the carcinoma stage due to MESP2 loss. Specifically, MESP2 suppressed formation of the TCF4/beta-catenin complex by competing with beta-catenin for binding to TCF4, which resulted in the inhibition of SKP2 transcriptional activation and subsequent p27 accumulation. GSEA further confirmed that the action of MESP2 as a suppressor of TCF4/beta-catenin activity further regulated SKP2 /p27 signalling in GC.

Interestingly, aside from MESP2, another bHLH transcription factor, ITF2, was also ascertained to inhibit activation of the TCF4/beta-catenin transcriptional complex. Different from MESP2, ITF2 directly interacted with beta-catenin, thereby reducing the binding between TCF4 and beta-catenin [13]. These findings indicated that the different locations of the bHLH domains of MESP2 and ITF2 led to the differences in their biomolecular mechanisms. Of note, MESP2 was not co-precipitated with the TCF4/beta-catenin complex, which signified that TCF4, beta-catenin, and MESP2 did not form a trimeric complex. Considering the observation of MESP2 in the cytoplasm and nucleus of GC cells, we have a particularly compelling need for further research, as it remains to be determined whether the anti-GC activity of MESP2 depends on its abilities to inhibit the TCF4/beta-catenin complex formation in the cytoplasm or on dissociation in the nucleus.

**Fig. 8:**
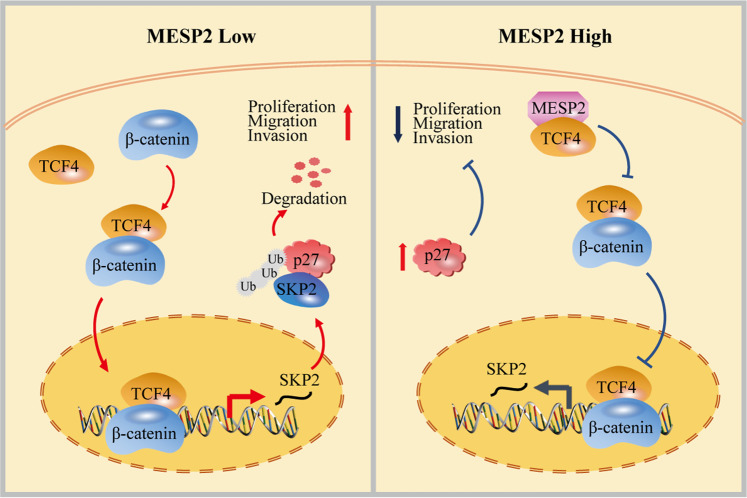
Hypothesis diagram illustrates the mechanisms of MESP2 roles in GC. MESP2 and beta-catenin competitively bind to TCF4, which suppresses GC progression by regulating the SKP2/p27 axis.

In summary, we report that MESP2 functions as a completely new tumor suppressor in GC. Mechanistically, MESP2 competes with beta-catenin to bind to TCF4, and blocks TCF4/beta-catenin complex transcriptional activity on the SKP2 promoter, which in turn, enhances p27 stability and expression levels through SKP2 suppression (Fig. 8). Accordingly, this study reveals MESP2 as a novel player in TCF4/beta-catenin signalling. We believe that this is the first comprehensive characterization of MESP2 role and mechanism in GC cells, implying that MESP2 is a prospective therapeutic target for patients with GC.

## Materials and methods

### Cell lines and cultures

All cell lines (HGC-27, MKN-45, SGC-7901, BGC-823, GES-1 and 293FT) were derived from American Type Culture Collection (ATCC, Beijing, China) and were cultured as described [31]. All cell lines tested negative for mycoplasma.

### Protein extraction, western blotting, and co-immunoprecipitation (co-IP) assays

RIPA buffer, supplemented with protease and phosphatase inhibitors, was utilized in cell lysis. Then sediments were removed by centrifugation at 12,000 rpm and 4 °C for 10 min, and the supernatants were separated for western blot experiments. The Nuclear and Cytoplasmic Protein Extraction Kit (Beyotime, Shanghai, China) was utilized to analyze protein distribution. 30 µg of protein were used in equal amounts for western blot experiments. For co-IP assays, cell lysates with specific antibodies were incubated on a turn table at 4°C overnight. Then, Protein A/G Agarose (Santa Cruz, Dallas, Texas, USA) was added for antibody attachment and the immunoprecipitants were boiled for SDS-PAGE. Details about the antibodies used in this study are described in Supplementary Table 1.

### immunohistochemical (IHC) staining

Embedded tumor tissue sections in paraffin were probed with MESP2 primary antibody (1:200, #bs-18796R, BIOSS, Beijing, China) or Ki67 primary antibody (1:200, #27309-1-AP, Proteintech, Wuhan, China), then tested as described [32, 33].

### RNA extraction and qRT-PCR

Total RNA was extracted using TRIzoL as previously described [34]. NanoDrop ND-2000 instrument (Yeasen, Shanghai, China) was used for RNA concentration measurement. GoScript™ Reverse Transcription System Kit (Promega, Beijing, China) was used for complementary DNA synthesis. qRT-PCR was used to examine cell mRNA levels. Details about the primers used in qRT-PCR are described in Supplementary Table 2.

### shRNAs and Plasmids or lentiviruses for transfection or infection

shMESP2, shSKP2, and shGFP were purchased from Sangon and cloned into the pLKO.1 vector. Sequences of all the shRNAs and plasmids used are provided in Supplementary Table 3. Lentiviruses were used for transfection or infection as described [35].

### Cell proliferation, migration, and invasion assays

As previously mentioned, 3-(4,5-dimethylthiazol-2-yl)-2,5-diphenylte-trazolium bromide (MTT) assays and bromodeoxyuridine (BrdU) staining were used to assess cell proliferation [34]. Migration, invasion, and wound healing assays were used for assessing the migratory and invasive behaviors of cells as described [35].

### Patient data analysis and patient tumor tissues

Patient data and gene expression data sets were obtained from R2: microarray analysis and visualization platform (http://hgserver1.amc.nl/cgibin/r2/main.cgi). Kaplan–Meier analysis and survival curves were carried out by using GraphPad Prism (version 6.0, GraphPad Software, San Diego, CA, USA). All cutoff values for separating high and low expression groups were determined by the online R2 database algorithm. Primary tumor specimens and prior approval were gained from the Ethics Committee of Daping Hospital (Chongqing, China). Tissue analysis was approved by the Ethics Committee of the Southwest University of China. All the patients provided written informed consent to participate.

### Animal studies

All animal experiments were permitted by the Animal Care and Use Committee of Southwest University and carried out in accordance with the Animal Care and Use Guidelines (Ministry of Science and Technology, Beijing, China). Five-week-old female nude mice were purchased and were raised in SPF room for a week to adapt to the new environment.

Subcutaneous xenotransplantation. Human GC lines (HGC-27) cells (1 × 10^5^ cells) were stably transfected with shGFP or shMESP2, grown in 100 μl medium (mixed with Matrigel at a 1:1 ratio), and injected hypodermically into the mice using a 1 ml Hamilton microliter syringe. Six female nude mice were used in each group. After euthanizing the mice, the tumors were removed, photographed, and weighed.

In vivo metastasis assay. Five million HGC-27 cells that stably expressing shGFP or shMESP2 were injected into the lateral tail vein of 5-week-old female nude mice. Six female nude mice were used in each group. All mice were raised 25 days in SPF room and finally killed by cervical dislocation. All lung samples were collected.

### Ubiquitination assay

The 293FT cells were co-transfected with relevant plasmid for 48 h. Then cells were dealt with MG-132 (50 μg/mL) for 6 h. Western blot and co-IP assays were performed after cell lysis.

### Expression microarray profiling

Total RNAs were extracted from shGFP- and shMESP2-transfected HGC-27 cells using TRIzoL reagent. All samples were analyzed by Sangon (Shanghai, China) according to mRNA expression microarray protocols.

### GSEA and GO analysis

GSEA analysis was performed by folding change data from differential expression analysis into GSEA software (Broad Institute). GO analysis was performed with marker genes from each subcluster by tools from DAVID.

The gene expression datasets (GSE15459 and GSE34942) analyzed in this study were obtained from the GEO database (https://www.ncbi.nlm.nih.gov/geo/). GSE15459 and GSE34942 was based on platform GPL570 ([HG-U133_Plus_2] Affymetrix Human Genome U133 Plus 2.0 Array). All of the data were freely available online, and this study did not involve any experiment on humans or animals performed by any of the authors.

### Proximity ligation assay technique

A proximity ligation assay was performed using Duolink^®^ Proximity Ligation Assay reagents according to the supplier’s guidelines (Sigma-Aldrich, Shanghai, China). Overexpred Flag-Mesp2 cells were cultured on the sliver. The cells were washed twice with 1 ml of PBS and fixed with 4% paraformaldehyde. Then perforated with 0.3% Triton solution. After sealed with 10% goat serum (purchased by Beyotime, Shanghai, China), Paired combinations of mouse (anti-Flag,Proteintech, 66008-3-Ig, 1:50) and rabbit (anti-TCF4, Proteintech, 13838-1-AP, 1:50) antibodies were incubated with the samples for overnight at 4 °C temperature. Proximity ligation assay minus and PLA plus probes (containing the secondary antibodies conjugated with oligonucleotides) were added and incubated 2 h at 4 °C. Afterwards, further oligonucleotides are added, allowed to hybridise to the PLA probes, and ligase joins the two hybridised oligonucleotides to a closed circle. The DNA is then amplified, and detection of the amplicons was carried out using the Duolink® In Situ Red detection kit (including Hoechst 33342 dye nuclear staining were mounted with Vectashield mounting media), resulting in red fluorescence signals.

### Chromatin immunoprecipitation

GC cells were cultured in 10 cm culture dish followed by 1% formaldehyde treatment for 10 min. Cells were pelletized and a supersonic device was used for shearing DNA into fragments between 200 and 500 bps. After chromatin sonication and centrifugation, 2 µg of Flag antibody (#8146, CST, Shanghai, China) or normal IgG were added overnight. The precipitated DNA was analyzed via qRT-PCR and the primers used are listed in Supplementary Table 4.

### Luciferase reporter assay

The SKP2 promoter and TCF-reporter plasmid (TOP Flash and FOP Flash) were subcloned into pGL-basical vector (Promega), and 293FT cells were co-transfected with luciferase reporter and pGL-TK reporter for 48 h. The luciferase activity was measured using a Dual-Luciferase Reporter Assay kit (YEASEN, Shanghai, China) according to the manufacturer’s instructions at 24 h post-transfection.

### Statistical analysis

All experiments were carried out with three technical and biological replicates. The statistical analysis was performed using GraphPad Prism. The Student’s t test or one-way ANOVA was used to analyze the significance between two or more groups. The data were presented as the means ± standard deviation (SD) of three independent experiments, where p < 0.05 was considered statistically significant (*p < 0.05, **p < 0.01, ***p < 0.001, ****p < 0.0001).

## Supplementary information

Supplementary Figure 1

Supplementary Figure 2

Supplementary figure legends

Supplementary Tables

## Data Availability

The datasets generated during and/or analysed during the current study are available from the corresponding author on reasonable request.

## References

[1] Padmanabhan N, Ushijima T, Tan P (2017). How to stomach an epigenetic insult: the gastric cancer epigenome. Nat Rev Gastroenterol Hepatol.

[2] Guo X, Lv X, Ru Y, Zhou F, Wang N, Xi H (2020). Circulating exosomal gastric cancer-associated long noncoding RNA1 as a biomarker for early detection and monitoring progression of gastric cancer: a multiphase study. JAMA Surg.

[3] McLean MH, El-Omar EM (2014). Genetics of gastric cancer. Nat Rev Gastroenterol Hepatol.

[4] Yabe T, Hoshijima K, Yamamoto T, Takada S (2016). Quadruple zebrafish mutant reveals different roles of Mesp genes in somite segmentation between mouse and zebrafish. Development.

[5] Dunty WC, Biris KK, Chalamalasetty RB, Taketo MM, Lewandoski M, Yamaguchi TP (2008). Wnt3a/beta-catenin signaling controls posterior body development by coordinating mesoderm formation and segmentation. Development.

[6] Rallis C, Pinchin SM, Ish-Horowicz D (2010). Cell-autonomous integrin control of Wnt and Notch signalling during somitogenesis. Development.

[7] Vaquerizas JM, Kummerfeld SK, Teichmann SA, Luscombe NM (2009). A census of human transcription factors: function, expression and evolution. Nat Rev Genet.

[8] Murre C, McCaw PS, Vaessin H, Caudy M, Jan LY, Jan YN (1989). Interactions between heterologous helix-loop-helix proteins generate complexes that bind specifically to a common DNA sequence. Cell.

[9] Torres-Machorro AL (2021). Homodimeric and heterodimeric interactions among vertebrate basic helix-loop-helix transcription factors. Intl J Mol Sci.

[10] de Martin X, Sodaei R, Santpere G (2021). Mechanisms of binding specificity among bHLH transcription factors. Int J Mol Sci.

[11] Tarczewska A, Greb-Markiewicz B (2019). The significance of the intrinsically disordered regions for the functions of the bHLH transcription factors. Int J Mol Sci.

[12] Zhang X, Liu R, Zhao N, Ji S, Hao C, Cui W (2019). Sohlh2 inhibits breast cancer cell proliferation by suppressing Wnt/β-catenin signaling pathway. Mol Carcinog.

[13] Shin H-W, Choi H, So D, Kim Y-I, Cho K, Chung H-J (2014). ITF2 prevents activation of the β-catenin-TCF4 complex in colon cancer cells and levels decrease with tumor progression. Gastroenterology.

[14] Ishiguro H, Wakasugi T, Terashita Y, Sakamoto N, Tanaka T, Sagawa H (2016). Nuclear expression of TCF4/TCF7L2 is correlated with poor prognosis in patients with esophageal squamous cell carcinoma. Cell Mol Biol Lett.

[15] Ma L, Lin K, Chang G, Chen Y, Yue C, Guo Q (2019). Aberrant activation of β-Catenin signaling drives Glioma Tumorigenesis via USP1-mediated stabilization of EZH2. Cancer Res.

[16] Hua F, Shang S, Yang Y-W, Zhang H-Z, Xu T-L, Yu J-J (2019). TRIB3 interacts with β-Catenin and TCF4 to increase stem cell features of colorectal cancer stem cells and tumorigenesis. Gastroenterology.

[17] Meyer SE, Muench DE, Rogers AM, Newkold TJ, Orr E, O’Brien E (2018). miR-196b target screen reveals mechanisms maintaining leukemia stemness with therapeutic potential. J Exp Med.

[18] Pérez-Luna M, Aguasca M, Perearnau A, Serratosa J, Martínez-Balbas M, Jesús Pujol M (2012). PCAF regulates the stability of the transcriptional regulator and cyclin-dependent kinase inhibitor p27 Kip1. Nucleic Acids Res.

[19] Huang H-Y, Kang H-Y, Li C-F, Eng H-L, Chou S-C, Lin C-N (2006). Skp2 overexpression is highly representative of intrinsic biological aggressiveness and independently associated with poor prognosis in primary localized myxofibrosarcomas. Clin Cancer Res: Off J Am Assoc Cancer Res.

[20] Razavipour SF, Harikumar KB, Slingerland JM (2020). p27 as a transcriptional regulator: new roles in development and cancer. Cancer Res.

[21] Jeannot P, Nowosad A, Perchey RT, Callot C, Bennana E, Katsube T (2017). p27 promotes invadopodia turnover and invasion through the regulation of the PAK1/Cortactin pathway. eLife.

[22] Bencivenga D, Caldarelli I, Stampone E, Mancini FP, Balestrieri ML, Della Ragione F (2017). p27 and human cancers: A reappraisal of a still enigmatic protein. Cancer Lett.

[23] Greb-Markiewicz B, Kazana W, Zarębski M, Ożyhar A (2019). The subcellular localization of bHLH transcription factor TCF4 is mediated by multiple nuclear localization and nuclear export signals. Sci Rep.

[24] Abbas M, Habib M, Naveed M, Karthik K, Dhama K, Shi M (2017). The relevance of gastric cancer biomarkers in prognosis and pre- and post- chemotherapy in clinical practice. Biomed Pharmacother.

[25] Oginuma M, Niwa Y, Chapman DL, Saga Y (2008). Mesp2 and Tbx6 cooperatively create periodic patterns coupled with the clock machinery during mouse somitogenesis. Development.

[26] Liang Q, Xu C, Chen X, Li X, Lu C, Zhou P (2015). The roles of Mesp family proteins: functional diversity and redundancy in differentiation of pluripotent stem cells and mammalian mesodermal development. Protein cell.

[27] Traub F, Mengel M, Lück HJ, Kreipe HH, von Wasielewski R (2006). Prognostic impact of Skp2 and p27 in human breast cancer. Breast Cancer Res Treat.

[28] Shen L, Qu X, Li H, Xu C, Wei M, Wang Q (2018). NDRG2 facilitates colorectal cancer differentiation through the regulation of Skp2-p21/p27 axis. Oncogene.

[29] Sonoda H, Inoue H, Ogawa K, Utsunomiya T, Masuda T-A, Mori M (2006). Significance of skp2 expression in primary breast cancer. Clin Cancer Res: Off J Am Assoc Cancer Res.

[30] Su J, Su B, Xia H, Liu F, Zhao X, Li J (2019). RORα suppresses Epithelial-to-Mesenchymal transition and invasion in human gastric cancer cells via the Wnt/β-Catenin pathway. Front Oncol.

[31] Yin C, Ke X, Zhang R, Hou J, Dong Z, Wang F (2019). G9a promotes cell proliferation and suppresses autophagy in gastric cancer by directly activating mTOR. FASEB J: Off Publ Fed Am Soc Exp Biol.

[32] He Q, Lin Z, Wang Z, Huang W, Tian D, Liu M (2020). SIX4 promotes hepatocellular carcinoma metastasis through upregulating YAP1 and c-MET. Oncogene.

[33] Du J, Dong Z, Tan L, Tan M, Zhang F, Zhang K (2020). Tubeimoside I inhibits cell proliferation and induces a partly disrupted and cytoprotective autophagy through rapidly hyperactivation of MEK1/2-ERK1/2 cascade via promoting PTP1B in Melanoma. Front Cell Dev Biol.

[34] Huang MY, Xuan F, Liu W, Cui HJ (2017). MINA controls proliferation and tumorigenesis of glioblastoma by epigenetically regulating cyclins and CDKs via H3K9me3 demethylation. Oncogene.

[35] Hou J, Deng Q, Zhou J, Zou J, Zhang Y, Tan P (2017). CSN6 controls the proliferation and metastasis of glioblastoma by CHIP-mediated degradation of EGFR. Oncogene.

