# Supplementary figures and images for: MESP2 binds competitively to TCF4 to suppress gastric cancer progression by regulating the SKP2/p27 axis
Source: Cell Death Discov. 2023 Mar 1;9:79. doi: 10.1038/s41420-023-01367-4 (PMC9975210; doi:10.1038/s41420-023-01367-4)

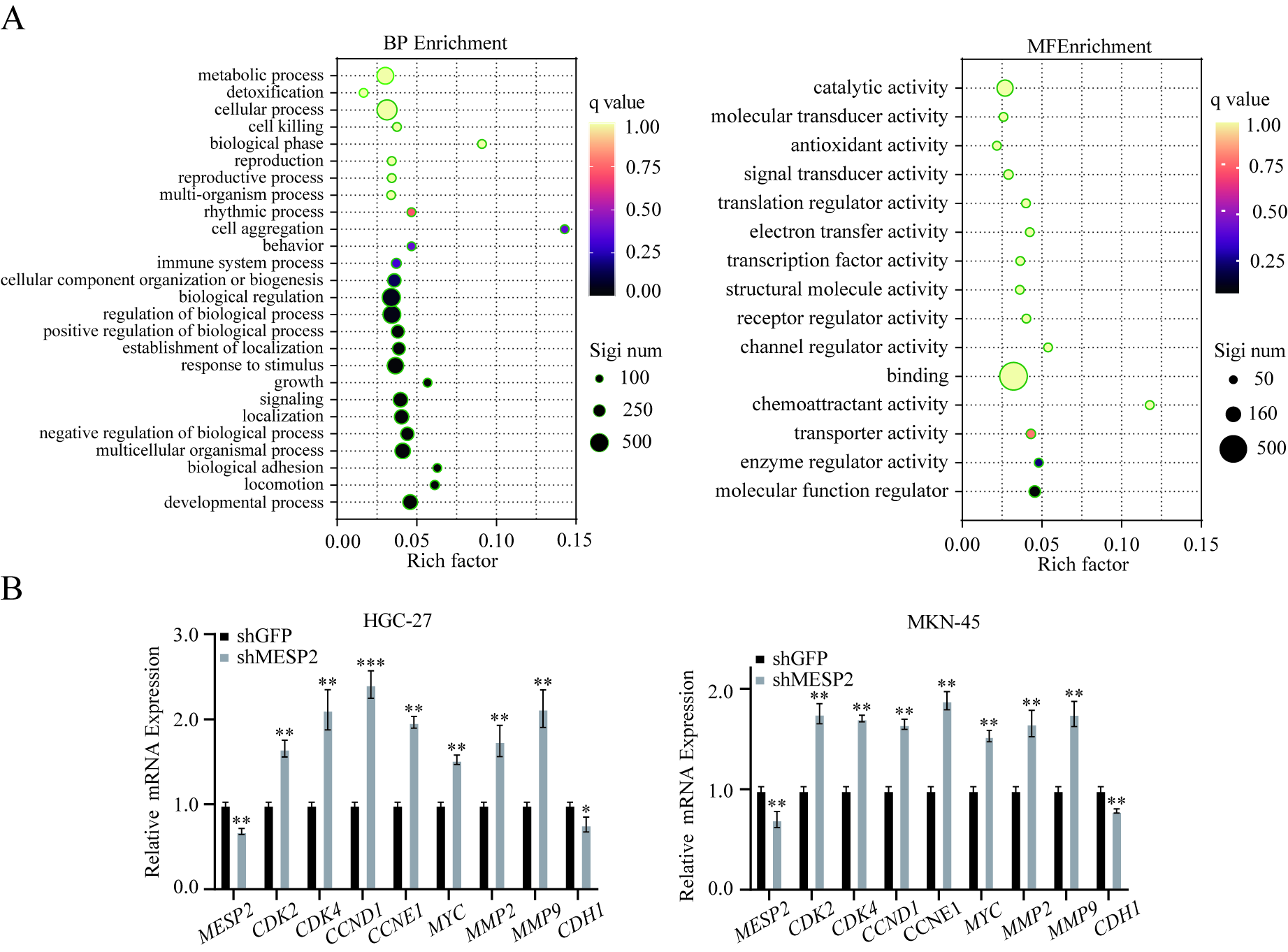

Supplement: Supplementary file 1 — Supplementary Figure 1 [file 41420_2023_1367_MOESM1_ESM.tif]

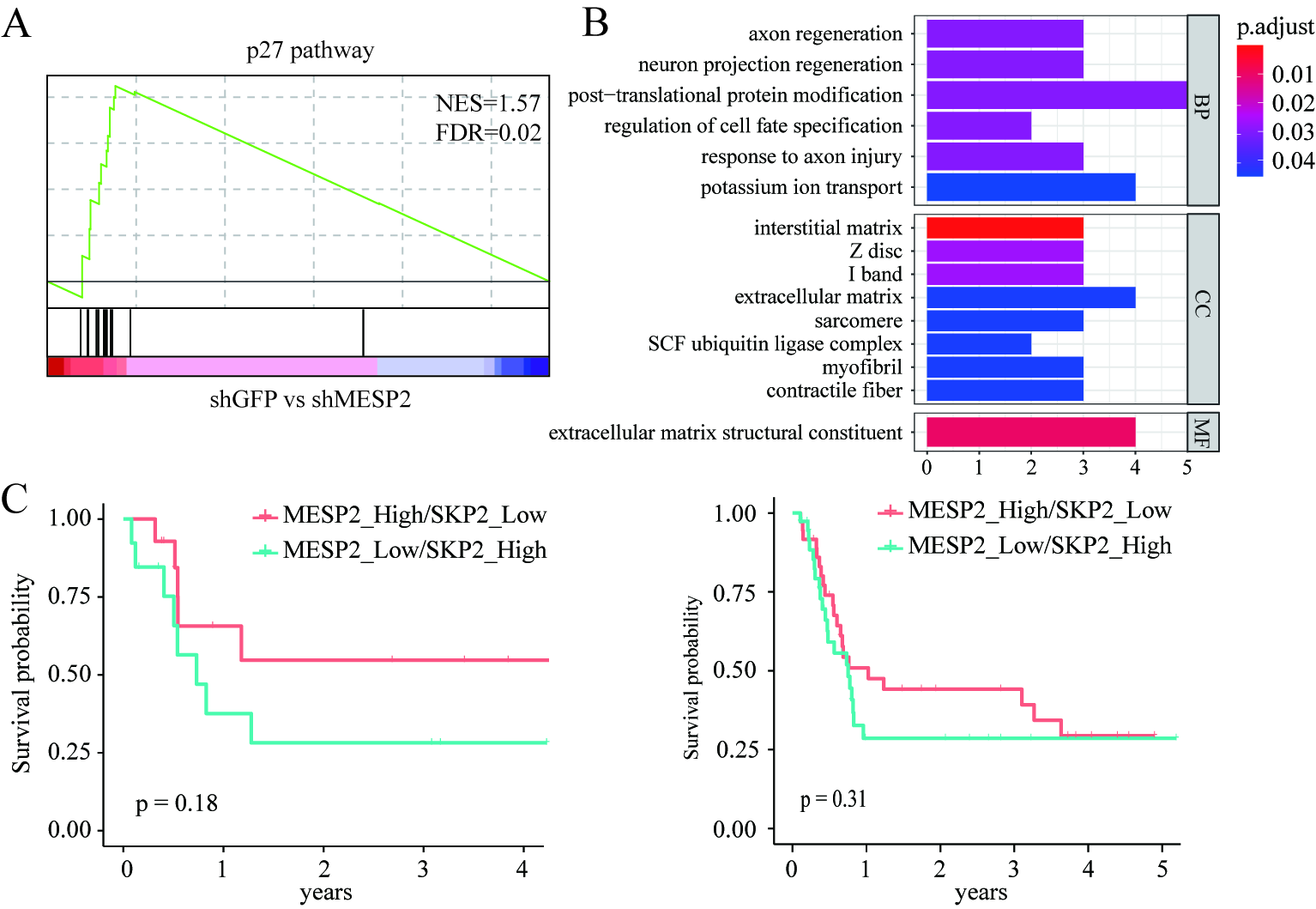

Supplement: Supplementary file 2 — Supplementary Figure 2 [file 41420_2023_1367_MOESM2_ESM.tif]
