# Supplementary material for: MESP2 binds competitively to TCF4 to suppress gastric cancer progression by regulating the SKP2/p27 axis
Source: Cell Death Discov. 2023 Mar 1;9:79. doi: 10.1038/s41420-023-01367-4 (PMC9975210; doi:10.1038/s41420-023-01367-4)
Supplement: Supplementary file 3 — Supplementary figure legends [file 41420_2023_1367_MOESM3_ESM.docx]

**Supplementary Figure 1**

**a.** Gene ontology (GO) enrichment analysis of differentially expressed genes in MESP2-knockdown HGC-27 cells. Biological process (left) and molecular function (right) enrichments. **b.** qRT-PCR detection of mRNA levels of genes associated with cell cycle and metastasis in MESP2-knockdown HGC-27 and MKN-45 cells. Data shown are means ± SD, n = 3, *P < 0.05, **P < 0.01, ***P < 0.001. **c.** The percentage of Mesp2, Ki67, P27 -positive staining was quantified. Data shown are means ± SD, n = 3, *P < 0.05, **P < 0.01, ***P < 0.001.

**Supplementary Figure 2**

**a.** GSEA of shGFP and shMESP2 groups, revealing the p27 signalling pathway enrichment score in HGC-27 cells. **b.** Gene ontology (GO) enrichment analysis of differentially expressed genes in MESP2-knockdown HGC-27 cells and the gene expression datasets (GSE15459 and GSE34942). BP: Biological process, CC: cellular component, MF: molecular function. **c.** Kaplan-Meier OS curves for the patients stratified by a combination of Mesp2 and skp2 in GC (left pane: Tumor Gastric (Batch B) - Tan - 56 - MAS5.0 - u133p2, right panel: Tumor Gastric - Tan - 192 - MAS5.0 - u133p2).
