# Supplementary material for: MESP2 binds competitively to TCF4 to suppress gastric cancer progression by regulating the SKP2/p27 axis
Source: Cell Death Discov. 2023 Mar 1;9:79. doi: 10.1038/s41420-023-01367-4 (PMC9975210; doi:10.1038/s41420-023-01367-4)
Supplement: Supplementary file 4 — Supplementary Tables [file 41420_2023_1367_MOESM4_ESM.docx]

Supplementary Table 1 Antibodies

| Antibodies | Source and Catalog number | Dilution |
| --- | --- | --- |
| Anti-MESP2 | Novus, NBP2-37424 | 1:1000 |
| Anti-TCF4 | Proteintech, 13838-1-AP | 1:1000 |
| Anti-p27 | Proteintech,25614-1-AP | 1:1000 |
| Anti-SKP2 | Proteintech,15010-1-AP | 1:1000 |
| Anti-β-catenin | Proteintech,66379-1-Ig | 1:1000 |
| Anti-CDK2 | Proteintech,10122-1-AP | 1:1000 |
| Anti-CDK4 | Proteintech,11026-1-AP | 1:1000 |
| Anti-cyclinD | Proteintech,26939-1-AP | 1:1000 |
| Anti-cyclinE | Proteintech,11554-1-AP | 1:1000 |
| Anti-E-cadherin | Proteintech,20874-1-AP | 1:1000 |
| Anti-N-cadherin | Proteintech,22018-1-AP | 1:1000 |
| Anti-MMP2 | Proteintech,10373-2-AP | 1:1000 |
| Anti-MMP9 | Proteintech,10375-2-AP | 1:1000 |
| Anti-HA tag | Proteintech,51064-2-AP | 1:1000 |
| Anti-Flag tag | Proteintech,20543-1-AP | 1:1000 |
| Anti-α-Tubuli­n | Proteintech,66031-1-Ig | 1:1000 |
| anti-Mouse IgG-HRP | beyotim, A0216 | 1:10000 |
| anti-Rabbit IgG-HRP | beyotim, A0208 | 1:10000 |
| anti-Rabbit IgG, Alexa Fluor 594 | Sigma-Aldrich, SAB4600407 | 1:1000 |
| anti-mouse IgG, Alexa Fluor 488 | Sigma-Aldrich, SAB4600407 | 1:1000 |
| anti-Rabbit IgG, Alexa Fluor 488 | Sigma-Aldrich, SAB4600389 | 1:1000 |
| anti-mouse IgG, Alexa Fluor 594 | Sigma-Aldrich, SAB4600321 | 1:1000 |

Supplementary Table 2 qRT-PCR primers

| Primers | Sequences |
| --- | --- |
| MESP2 | F: 5’-CACGACCACTGGATCTTCGC-3’  R: 5’-AACCCGACGAATCGGAGGA-3’ |
| TCF4 | F: 5’-GCCTCTTATCACGTACAGCAAT-3’  R: 5’-GCCAGGCGATAGTGGGTAAT-3’ |
| p27 | F: 5’-AACGTGCGAGTGTCTAACGG-3’  R: 5’-CCCTCTAGGGGTTTGTGATTCT-3’ |
| SKP2 | F: 5’-ATGCCCCAATCTTGTCCATCT-3’  R: 5’-CACCGACTGAGTGATAGGTGT-3’ |
| CTNNB1 | F: 5’-CATCTACACAGTTTGATGCTGCT-3’  R: 5’-GCAGTTTTGTCAGTTCAGGGA-3’ |
| CDK2 | F: 5’-CCAGGAGTTACTTCTATGCCTGA-3’  R: 5’-TTCATCCAGGGGAGGTACAAC-3’ |
| CDK4 | F: 5’-ATGGCTACCTCTCGATATGAGC-3’  R: 5’-CATTGGGGACTCTCACACTCT-3’ |
| CCND1 | F: 5’-GCTGCGAAGTGGAAACCATC-3’  R: 5’-CCTCCTTCTGCACACATTTGAA-3’ |
| CDH1 | F: 5’-CGAGAGCTACACGTTCACGG-3’  R: 5’-GGGTGTCGAGGGAAAAATAGG-3’ |
| MMP3 | F: 5’-GATACCCCTTTGACGGTAAGGA-3’  R: 5’-CCTTCTCCCAAGGTCCATAGC-3’ |
| MMP3 | F: 5’-CGGTTCCGCCTGTCTCAAG-3’  R: 5’-CGCCAAAAGTGCCTGTCTT-3’ |
| MMP9 | F: 5’-GGGACGCAGACATCGTCATC-3’  R: 5’-TCGTCATCGTCGAAATGGGC-3’ |
| GAPDH | F: 5’-GGAGCGAGATCCCTCCAAAAT-3’  R: 5’-GGCTGTTGTCATACTTCTCATGG-3’ |

Supplementary Table 3 shRNA and plasmids

| Primers/gene | Sequences/Gene ID | Vector |
| --- | --- | --- |
| shGFP | 5’-GCAAGCTGACCCTGAAGTTCA-3’ | pLKO.1 - Puro |
| shMESP2#1 | 5’-GCCGCCTTCCAGCTCAGTGAA-3’ | pLKO.1 - Puro |
| shMESP2#2 | 5’-TCCGCTACATCGGCCACCTAT-3’ | pLKO.1 - Puro |
| shSKP2#1 | 5’-GCCTAAGCTAAATCGAGAGAA-3’ | pLKO.1 - Puro |
| shSKP2#2 | 5’-CCATTGTCAATACTCTCGCAA-3 | pLKO.1 - Puro |
| MESP2 | Gene ID: 145873 | pCDH-GFP+Puro-3 x Flag-N |
| MESP2-bHLH | MESP2 (80-136aa) | pCDH-GFP+Puro-3 x Flag-N |
| HA-TCF4 | Gene ID: 6934 | pCDH-CMV-MCS-EF1-Puro-copGFP |
| HA-TCF4-β-cat BD | TCF4 (1-54 aa) | pCDH-CMV-MCS-EF1-Puro-copGFP |

Supplementary Table 4 ChIP experimental primers

| Primers | Sequences |
| --- | --- |
| SKP2-1 | F: 5’-TTCCAATCAAAACCAAGTTGTGAACAGA -3’  R: 5’-ACCATGCGAAATCCATTGGGCAAACTAT -3’ |
| SKP2-2 | F: 5’-TGGAATATGTCACGCTCCCTACTCTTAC -3’  R: 5’-TAAAATAACGCACAACAGCTAAACACGC -3’ |
| SKP2-3 | F: 5’-CTTCCTGGCAAAAGGACGAACAGGTGCG -3’  R: 5’-AGACGCTGTCTGGCGAGATCGGACGGTG -3’ |
| SKP2-4 | F: 5’-AGGAAGTAAGGGCGAGCCCGTCGGACTA -3’  R: 5’-AGGCCCGGGAGTTTAAAATACGTGCATT -3’ |
| SKP2-5 | F: 5’-GCCAATGGTTGGTCCATAAATGATCGTG -3’  R: 5’-GCTGAAGAGCAAAGGGAGTGACAAAGAC -3’ |
